# ‘A machine for recreating life’: an introduction to reproduction on film

**DOI:** 10.1017/S0007087417000632

**Published:** 2017-09

**Authors:** JESSE OLSZYNKO-GRYN, PATRICK ELLIS

**Affiliations:** * Department of History and Philosophy of Science, University of Cambridge, Free School Lane, Cambridge, CB2 3RH, UK. Email: jo312@cam.ac.uk.; ** School of Literature, Media, and Communication, Georgia Institute of Technology, 215 Bobby Dodd Way NW, Atlanta, GA 30332, USA. Email: pgellis@gatech.edu.

## Abstract

Reproduction is one of the most persistently generative themes in the history of science and cinema. Cabbage fairies, clones and monstrous creations have fascinated filmmakers and audiences for more than a century. Today we have grown accustomed not only to the once controversial portrayals of sperm, eggs and embryos in biology and medicine, but also to the artificial wombs and dystopian futures of science fiction and fantasy. Yet, while scholars have examined key films and genres, especially in response to the recent cycle of Hollywood ‘mom coms’, the analytic potential of reproduction on film as a larger theme remains largely untapped. This introduction to a special issue aims to consolidate a disparate literature by exploring diverse strands of film studies that are rarely considered in the same frame. It traces the contours of a little-studied history, pauses to consider in greater detail a few particularly instructive examples, and underscores some promising lines of inquiry. Along the way, it introduces the six original articles that constitute *Reproduction on Film*.

Reproductive metaphors abound in discussions of cinema. The ‘birth’ of cinema is typically dated to 1895. Like the other creative arts, cinema has its founding ‘fathers’ (the Lumière brothers, Thomas Edison) and ‘mothers’ (Alice Guy-Blaché). Like all cultural endeavours, filmmaking has been likened to a collaborative, creative enterprise akin to making babies. Film has allowed women and men to ‘give birth’ metaphorically, as directors, and literally, as actors. Films have a *preproduction* stage and like embryos go through *development*. The capacious and slippery term *reproduction* itself can refer to a biological and social process of human procreation, or to a mechanical process of copying images now closely associated with the Marxist philosopher Walter Benjamin's classic essay ‘The work of art in the age of mechanical reproduction’ (1935).^1^ This ambivalence is likewise found in the title of this introduction to a special issue, drawn as it is from one of the first documentaries devoted to the history of cinema, *A Machine for Recreating Life* (1924–1933).^2^ Cinema, it was thought, re-creates life both through its approximation to reality – its verisimilitude – and through its movement of the still image – its vivification.

Beyond metaphors and language, reproductive science, technology and medicine have developed especially since the early 1900s alongside techniques of capturing, displaying and analysing moving images. For over a century, embryologists, obstetricians and scientists and medical professionals of other kinds have made movies for purposes of research, teaching and propaganda. Meanwhile, the mass-market products of lucrative entertainment industries have made previously taboo reproductive experiences, practices and technologies more publicly visible than ever before. The road to *What to Expect When You're Expecting* (2012), the first Hollywood movie to be based on a pregnancy advice manual, has been winding and bumpy, with significant detours along the way. The canon of reproductive film includes some of the best-loved and most controversial artefacts in the history of the cinema. The relevant scholarship is extensive, but often organized by genre, analytically focused on the narrative content of fiction films – and the types of representation embedded therein – and scattered in disparate studies on a range of more discrete topics, including sex education, genetics and eugenics.^3^

Cinema, as we shall see, has always been reproductive. But with few exceptions film studies did not take much notice of reproduction until around 1990. In the United States, heavily mediatized landmark events, including the sensational ‘Baby M’ surrogacy case of the late 1980s and the appearance in 1991 of a visibly pregnant Demi Moore on the cover of *Vanity Fair*, contributed to an increasingly public culture of reproduction.^4^ Hollywood got in on the act with romantic comedies such as *Baby Boom* (1987), *Three Men and a Baby* (1987) and *Look Who's Talking* (1989) (Figure 1).^5^ Influential critiques of foetal imaging took shape at around the same time.^6^ So too did the feminist critique of representations of the maternal in cinema, for instance in Hollywood films that blamed mothers for ills that befell their children.^7^ More recent literary studies have probed the latest crop of IVF films, in which romance and marriage often follow (medically assisted) conception, rather than the other way around.^8^

**Figure 1.**
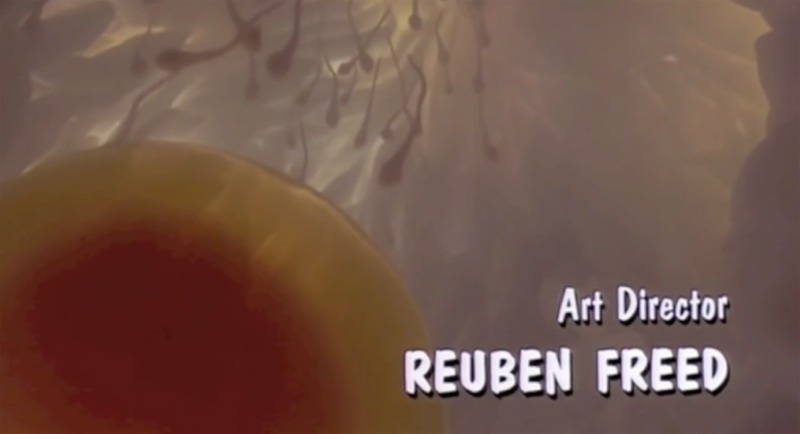
Screen capture from the memorable opening credits of Amy Heckerling's *Look Who's Talking* (1989) showing what appear to be human sperm racing towards an egg, but are in fact vinyl ‘sperm’ weighed down with fishing sinkers and filmed in a tank by an underwater camera. Produced by MCEG, distributed by Tri-Star Pictures.

Taking film – broadly conceived to include cinema, television, video and digital imaging – as the case study, this special issue explores the highly generative confluence of biological reproduction and mechanical reproduction. It also considers the production and circulation of moving images in relation to a broader visual and material culture of research, teaching and communication that has included – and continues to include – a diversity of publication formats, from three-dimensional models to illustrated textbooks, scientific journals and magazines, to name just a few.^9^ How did reproduction shape film and vice versa? *Reproduction on Film* will begin to answer this and other questions.

The six contributions to *Reproduction on Film* examine developments mainly in Britain and the United States, from around 1910 to the present. In this introduction we take a somewhat broader view, both geographically and chronologically. Beginning in the late nineteenth century, we first examine early genres and editing techniques that produced newborn babies from behind cabbage patches and out of thin air. Dwelling on the silent period, we consider Soviet avant-garde filmmaker Sergei Eisenstein's career-defining engagement with a foetus, and the impact of this encounter on his theory of animation. We next investigate the promises and constraints of biological and medical cinematography, particularly in relation to the manipulation of time and motion; embryologists used time-lapse techniques to accelerate otherwise imperceptibly slow developmental processes, while obstetricians learned how to pause their films in order to make rapid surgical manoeuvres intelligible to students. Turning to genres that blurred the boundaries between entertainment and education, we consider the shock of the birth scene in ‘exploitation’ and avant-garde cinema before concluding with a glance at the mainstreaming of reproduction in cinema as well as the related media of television, video and streaming. Overall, we propose that moving images not only have significantly shaped the scientific understandings, private experiences and public cultures of reproduction, but also have done so in media-specific ways.

## The birth of cinema and the cinema of birth

Prior to the ‘birth of cinema’ in 1895, a variety of nineteenth-century mechanical devices and display techniques, as well as music hall and fairground attractions, offered appeals similar to those that would soon become associated with the Lumière cinematograph, a portable hand-cranked device that served as both camera and projector: optical toys such as the zoetrope (the ‘wheel of life’) ‘animated’ the inanimate, panorama paintings immersed the viewer in another space, magic-lantern projectors were indispensable in medical education, and mechanical postcards added a kinetic movement to a simple message.^10^ This last format, the postcard, played a crucial role in the history of reproduction on film.^11^

**Figure 2.**
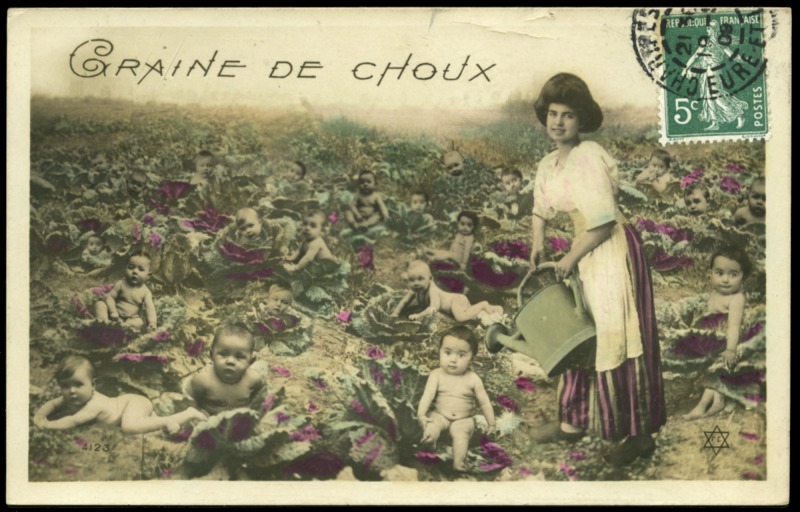
Typical example of the cabbage-patch baby motif of the French postcard (*c*.1900).

Postcards boomed in Europe and North America at the turn of the century, and among the most successful genres were birth announcements. Usually featuring imagery of storks bringing babies to new parents or (in the francophone world, where they were known as *faire-parts de naissance*) equally politely premised upon the folk notion of babies emerging from cabbage patches, they would be sent by new parents to friends and relations (Figure 2).^12^ Postcards were a common medium for adaptation in the first decade of film, given their abundance and affordable popularity at the time; adaptations of postcard landscapes, postcard humour, and postcard birth announcements were common.^13^ The most sustained engagement with pregnancy and birth on screen in the earliest part of film history was an adaptation of the birth-announcement genre of postcard, made by the pioneering French filmmaker Alice Guy-Blaché.

**Figure 3.**
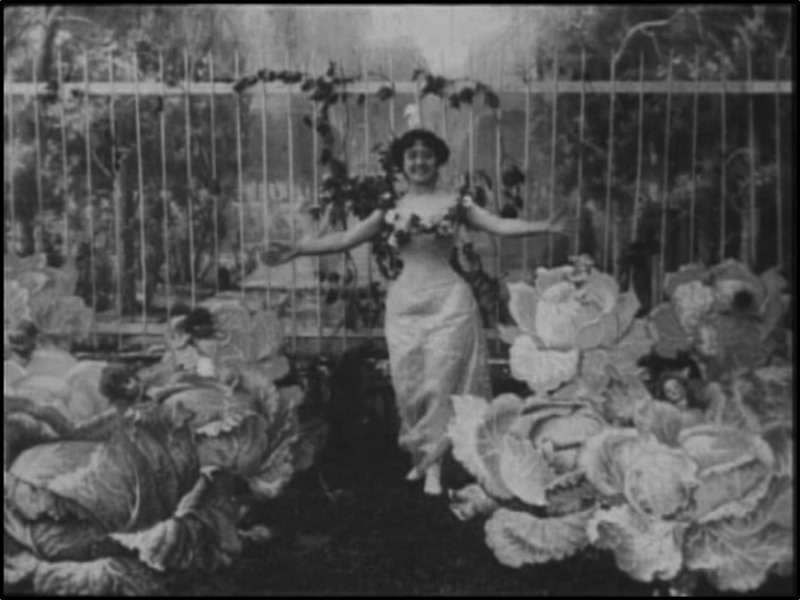
Screen capture from Alice Guy-Blaché’s *The Cabbage Fairy* (1896); note the dolls’ heads protruding from behind the cabbages. Produced by Gaumont.

*The Cabbage Fairy*, made in 1896 and barely one minute long, was a postcard brought to life (Figure 3). A non-theatrical ‘demonstration film’ intended to promote the Gaumont company's camera, it showed a fairy plucking newborns from a painted wooden cabbage patch and laying the babies at her feet. The film was a success, and (such was the technology of the time) had to be remade by Guy-Blaché twice in order to strike new prints.^14^ Perhaps as a result of this success, Guy-Blaché went on to make a number of films on reproductive topics, including *First Class Midwife* (1902), which survives, and *Their First Baby* (1911), now lost. None of this work was as unusual or as comic as *Madame Has Her Cravings* (1906), in which an insatiable pregnant woman is driven to steal increasingly inappropriate foodstuffs, including herring from a tramp and absinthe from a café. The film ends with her giving birth in a cabbage patch, in a trick cut.^15^

**Figure 4.**
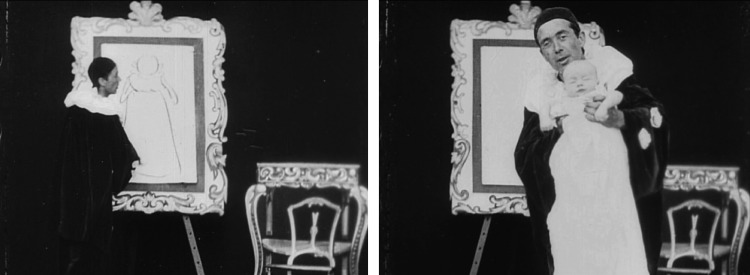
Consecutive screen captures from Walter Booth's *Artistic Creation* (1901), showing the ‘trick birth’ of a baby. Produced by Robert Paul's Animatograph Works.

Birth often occurred in a trick shot, a special effect invented and much deployed in the era of ‘early cinema’ (1895 to *c*.1913).^16^ To produce a trick shot the filmmaker either stops cranking the camera while it is filming, rearranges the scene (in this case adding an infant child), and starts the camera again, or, more commonly, cuts and glues pieces of the film together as a substitution splice in order to better conceal the trick.^17^ The alternative sources for children in early film are the flora and fauna of myth (the cabbage patch, the stork); or off screen, from the euphemism of implied space. To show a documentary birth outside a medical context would have been unthinkable. The trick cut let newborns be produced out of thin air. For example, in *Artistic Creation* (1901) the London trick filmmaker Walter Booth has a lightning sketch artist transform a drawn infant into living one in a single cut (Figure 4).^18^

Moral panic about venereal disease and racial degeneration resulted in the production during and after the First World War of numerous ‘social hygiene’ films. These included *S.O.S.: A Message to Humanity* (1917), a film about congenital syphilis and ‘eugenic marriage’, and *Whatsoever a Man Soweth* (1917), a British War Office production about a young Canadian soldier who learns of the ‘grave evils’ of syphilis.^19^ By then, an institutional ‘second birth’ of cinema was well under way, this time through the rise of the movie theatre.^20^ As cinema became increasingly dominant as an independent mass medium in its own right, older notions, such as the idea of ‘maternal impression’, were reworked in connection to moving images: a *Variety* critic worried that ‘vivid portraits of defectives’ in *The Black Stork* (1916), a highly publicized film that advocated eugenic infanticide, ‘would cause birth defects if pregnant women were allowed to see them’.^21^ Conversely, as Patrick Ellis shows in this issue, New York suffragette Electra Sparks advocated that poor, urban pregnant women should go to the movie theatre in order to form positive ‘mental pictures’ and so produce attractive, healthy children.

Some directors responded to moralizing propaganda with satirical films of their own, such as Edwin D. Porter's spoof of eugenics, *The Strenuous Life; or, Anti-race Suicide* (1904).^22^ *The Over-Incubated Baby* (1901) parodied the popular ‘incubator baby sideshow’, while *The Miraculous Waters* (1914), an Italian film, seemed to cheekily endorse infidelity as the ‘cure’ for a childless marriage after hydrotherapy fails to restore the husband's potency.^23^ In Japan and Soviet Russia, where abortion was made legal between 1920 and 1935, cinema was still less constrained by Christian morality. *Kid Commotion* (1935), a slapstick *benshi* or narrated ‘silent’ film originally titled *Birth (Out of) Control*, cast Shigeru Ogura, Japan's Charlie Chaplin, as an unemployed father and midwife for a day.^24^ Abram Room's *Bed and Sofa* (1927) humorously depicted a *ménage à trois* – a woman and two men – in a Moscow flat and frankly depicted a visit to a private abortion clinic.^25^

The Soviet avant-garde, too, found inspiration in reproduction. Dziga Vertov's experimental classic *Man with a Movie Camera* (1929), edited by his wife Yelizaveta Svilova, cross-cut documentary childbirth footage with that of a funeral procession.^26^ And Sergei Eisenstein, one of the most influential filmmakers and film theorists of the silent era, had a formative encounter with a human foetus while filming an educational film about abortion.

**Figure 5.**
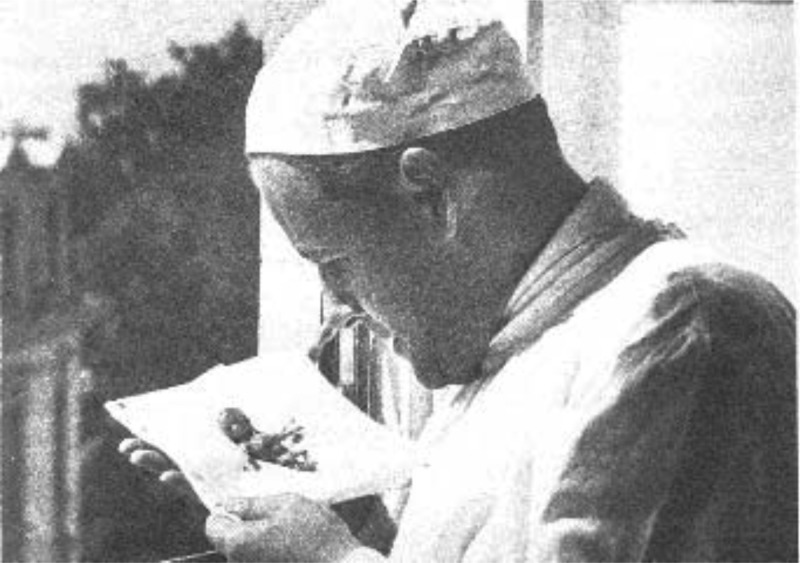
One of several photographs Sergei Eisenstein had taken of himself holding and contemplating a dying foetus at the Zurich women's clinic where *Frauennot-Frauenglück* (1929–1930) was filmed. Anne Nesbet, *Savage Junctures: Sergei Eisenstein and the Shape of Thinking*, London: I.B. Tauris, 2003, p. 140, courtesy of Anne Nesbet.

Celebrated for *Battleship Potemkin* (1925) and the development of montage theory, in his later years Eisenstein developed a devotion to Disney cartoons and a posthumously published theory of animation that corresponded to it.^27^ Writing about Disney, Eisenstein recalled visiting a women's clinic in Zurich in 1929, where he was filming *The Misery and Fortune of Women* (*Frauennot-Frauenglück*), a health education film that warned of the dangers of illegal, unprofessional abortion and promoted the wonders of hygienic medical childbirth, caesarean section, blood transfusion and surgical abortion.^28^ The experience of seeing ‘a little living being, dying in [his] hands in about ten minutes after its premature appearance in the world’ was profound, and he had himself photographed with the foetus (Figure 5).^29^ Eisenstein, as film scholar Anne Nesbet notes, later acquired a preserved foetus and kept it, as a ‘souvenir’ of his Zurich experience, in a jar on a bookshelf in his Moscow apartment.^30^

The experience was to inspire Eisenstein's theory of the plasmatic, which he defined as follows:

The rejection of the constraint of form, fixed once and for all, freedom from ossification, an ability to take on any form dynamically. An ability which I would call ‘plasmaticity’, for here a being, represented in a drawing, a being of a given form, a being that has achieved a particular appearance, behaves itself like primordial protoplasm, not yet having a stable form, but capable of taking on any and all forms of animal life on the ladder of evolution.^31^

In application, he meant the metamorphic quality of animation – the ‘stretch and squash’ that characters performed, the water-balloon physiology of the dwarfs in *Snow White and the Seven Dwarfs* (1937). For Eisenstein, the unfixed cartoon character was full of foetal potential. He imagined animation itself as a kind of quickening, bringing the non-living to life, animating the inanimate. This understanding of animation, prompted by the encounter between a Soviet filmmaker and a Swiss foetus in 1930, persists in the literature to this day.^32^

## Embryological and obstetric filmmaking

Movement, proclaimed Anthony Michaelis in his encyclopedic *Research Films in Biology, Anthropology, Psychology, and Medicine*, ‘is one of the characteristics of life: it begins with the penetration of the ovum by the spermatozoon and ends with last cytoplastic streaming of the cells. Both have been registered on motion picture film’.^33^ For Michaelis, the greatest contribution of cinematography to biology was its ability to produce quantifiable records of movement ‘on any given scale of time and length’. In the hands of an embryologist, time-lapse cinematography became the ‘perfect technique’ for recording the ‘slowly growing embryo’. The difficulty, as Michaelis saw it, lay not in the mechanical limitations of the cine camera, but in the biological properties of living organisms; only when the living embryo could be rendered transparent and its extraneous movements prevented – without disturbing the natural movements in the course of development – would it ‘become equally possible to record all its growth stages by means of time-lapse cinemicrography’.^34^

**Figure 6.**
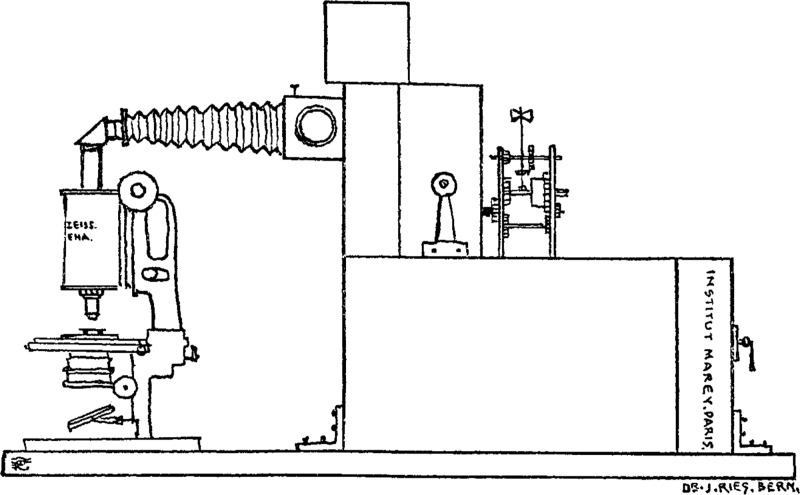
Line drawing of the hybrid apparatus used by Ries to make the first time-lapse films of sea urchin fertilization and development. Julius Ries, ‘Kinematographie der Befruchtung und Zellteilung’, *Archiv für Mikroskopische Anatomie und Entwicklungsgeschichte* (1909) 74, pp. 1–31, 3, with permission of Springer.

Beginning in the late nineteenth century, embryologists created and consulted standardized images of ‘normal’ embryological development for a range of invertebrate and vertebrate species. Organized into stages, series and tables, these visual standards organized a discipline and have sustained developmental biology to this day.^35^ At around the same time, French physiologist Etienne-Jules Marey and British American photographer Eadweard Muybridge finessed the method of ‘chronophotography’ for visualizing human and animal motion in a series or grid of still images.^36^ In 1908, Swiss biologist Julius Ries filmed urchin development at the Marey Institute in Paris with a Lumière cinematograph connected via a prism to a Zeiss microscope (Figure 6).^37^ Though far from the coast, he was able to obtain fresh specimens from Les Halles, the central food market of Paris. Starting with a single, fertilized egg, Ries recorded the first hours in the life of the organism. Microcinematography enabled him to visualize developmental change that was too slow to directly perceive under the microscope. ‘One really believes one has a living, developing egg before one’, he remarked of his achievement.^38^

**Figure 7.**
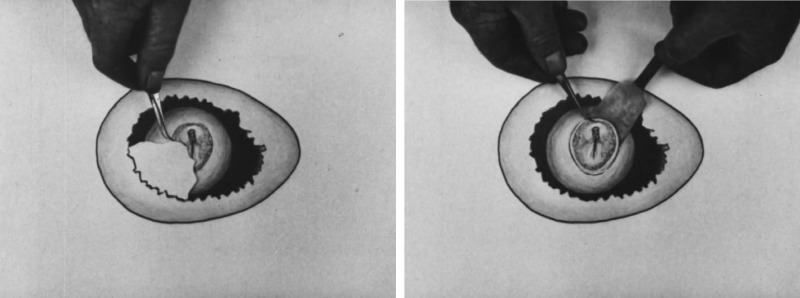
Screen captures from Bradley Patten and Theodore Kramer's *Development of a Bird Embryo* (1934), showing the removal of a piece of eggshell followed by that of a ‘living chick embryo from the egg’. This animated sequence comes after live-action footage of the same process. The British Medical Association's copy of the film is available online at http://catalogue.wellcomelibrary.org/record=b1672552~S3.

In contrast to sea urchins and other invertebrates, vertebrate embryos posed additional challenges for would-be cinematographers, including obtaining rarer and more fragile specimens as well as keeping them alive during often lengthier periods of development. *The Development of the Fertilized Rabbit's Ovum* (1929), a celebrated time-lapse film by American embryologist Warren H. Lewis, compressed seventy-two hours of development into fourteen minutes of projection time.^39^ By the 1940s embryologists had filmed development in amphibians, incubated chick embryos, mice, rabbits, worms and zebra fish.^40^ Today, as Janina Wellmann discusses in this issue, systems biologists are attempting to create a ‘digital embryo’, a computational model that enables the visualization of development at the cellular level in real time. In the decades before digital technologies, however, embryological films routinely employed intertitles, animated drawings and staged documentary footage (of specimen preparation and filmmaking) to elucidate and supplement the elusive process of development (Figure 7).

Magnified, moving images of embryos revealed much that was previously hidden, but there were limits to how much manipulation an embryo could stand. The later stages of mammalian development were particularly resistant to filming. As for human embryos, the additional, specific challenge of sourcing foetal material (from abortions or miscarriages) ‘precluded any films of their developmental stages’.^41^ American anatomist Davenport Hooker, however, did manage to film the reflexive reactions of dying aborted human foetuses. Today, the most remarkable feature of the films Hooker made between 1932 and 1963 is just how uncontroversial they were. *Time* magazine favourably reported on Hooker's research on ‘living abortuses’ in 1938.^42^

In contrast to the developing embryo, the process of delivery and childbirth was comparatively amenable to medical filmmaking. In this issue Caitjan Gainty examines the efforts, in the late 1920s, of Chicago obstetrician Joseph DeLee to transform the birthing room into a film studio, as well as his later objections to *The Fight for Life* (1940), a documentary about DeLee's own maternity ward. DeLee's approach was highly distinctive, not least because of the way he extended the engineering concept of ‘streamlining’ to childbirth, but he was not the first obstetrician to take up cinematography. A decade earlier, Paris obstetrician Victor Wallich had produced a series of short obstetric teaching films.^43^

**Figure 8.**
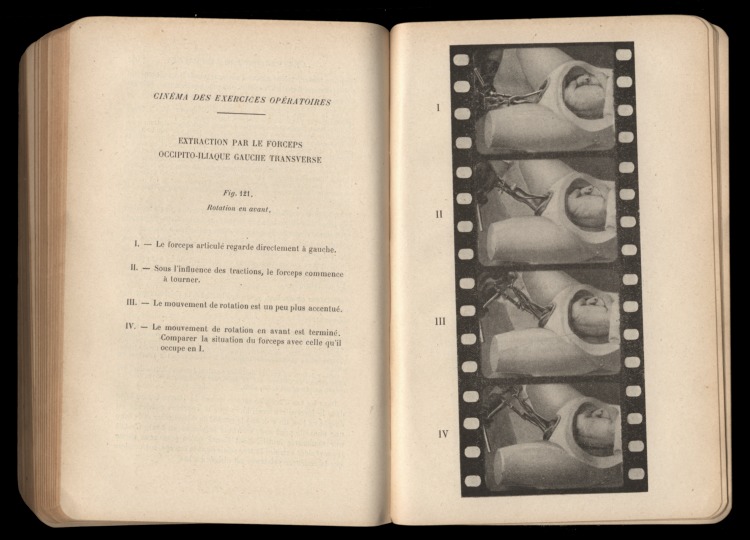
The fourth edition of Victor Wallich's textbook, *Eléments d'obstétrique*, Paris: Masson, 1921, pp. 530–531, open to pages picturing and describing four frames of a surgical manoeuvre with forceps.

In 1916 Wallich recorded various interventions, including forceps delivery, performed on a mannequin, which, in contrast to the living patient, enabled the interior movements of the operations to be visualized. Later editions of his popular textbook, *Eléments d'obstétrique*, were illustrated with enlarged photographs of short strips of film to show in a few frames the stages of an operative movement.^44^ Obstetric models, textbooks and cinematography thus came together on a single page (Figure 8).^45^ Wallich further used his films for teaching, screening them to students using an innovative stop-frame device to pause the action without causing the highly flammable nitrate film stock to burn up under the heat of the projection lamp. Few universities, however, could afford expensive 35 mm film projectors and, besides, did not have access to Wallich's innovation for ‘pausing’ the action. Safety regulations and fire laws further inhibited the adoption of film in classrooms. So, despite the efforts of Wallich and other enthusiasts, the audience for teaching films remained limited by practical and financial constraints. Medical students were more likely to encounter obstetric cinematography second-hand, in textbooks.

The advent of portable and less expensive 16 mm projectors, as well as non-flammable celluloid film stocks, facilitated the instructional uses of films, especially after the Second World War.^46^ Medical films of reproduction and, of course, many other aspects of biology and other sciences increasingly circulated as universities created film libraries, catalogues and distribution networks. Medically approved ‘factual films’, such as *Human Reproduction* (1947) and Encyclopaedia Britannica's *Biography of the Unborn* (1956), combined documentary footage, animated drawings, X-ray photographs and models to depict intrauterine life. These were nominally produced for married parents, but other films were intended for more specialist audiences.^47^ *Birth and the First Fifteen Minutes of Life* (1947), which showed the ‘removal of the placenta’, was made available ‘only to advanced classes in psychology and medical students in groups under the leadership of a physician or a senior member of a psychological faculty’.^48^ In 1950 it could be rented for four dollars a day or purchased for seventy-five, significant sums of money in those days. Despite the barriers medical professionals erected, educational films were widely appropriated and found mass audiences through alternative distribution networks that persisted until the 1960s.

## From exploitation to avant-garde

Between 1930 and the late 1950s, as David A. Kirby demonstrates in this issue, American film censors treated the biological reality of human reproduction as sacred but horrific; they believed that pregnancy and childbirth should be celebrated but not seen, and feared that realistic portrayals would put young women off pursuing motherhood.^49^ They were only partly successful in their mission. Whereas major studios worked closely with the censors to ensure the widest possible distribution, an itinerant group of entrepreneurial roadshowmen produced and distributed cheaply made ‘exploitation’ films on prohibited topics, including reproduction.

So-called exploiteers appropriated and exhibited medical footage, including of childbirth, before general audiences in theatrical venues typically reserved for entertainment films. In so doing, they challenged the boundaries between educational and obscene visual materials that medical professionals, the film industry and censor boards worked hard to maintain. Exploitation films traded in forbidden spectacles and ‘bad taste’. Low production costs, alternative distribution networks and distinctive marketing techniques set them apart from major studio releases. These titillating films, which flourished at the height of the Hays Code, showcased nudity, sex, drugs, abortion and childbirth, but stopped short of hard-core pornography. In contrast to stag films, which were technically illegal, were always short (one or two reels), depicted actual non-stimulated sex, and were shown privately to men only, exploitation films were legal, often feature-length, and shown openly to mixed audiences.^50^

Whereas major studio releases typically opened with four hundred prints in the 1930s or 1940s, ‘exploiteers’ would strike no more than fifteen or twenty prints of any given film, but these could remain in circulation for decades.^51^ As film historian Eric Schaefer explains in his comprehensive survey of the genre,

Going to an exploitation film was often a carnival-like event because of the extrafilmic practices that accompanied the show. Lectures, slide presentations, the sale of pamphlets or books on the picture's topic, and the presence of uniformed ‘nurses’ to attend to those who might faint due to the ‘shocking’ sights became a major part of the exploitation film experience.^52^

As birth control became more socially acceptable in the 1930s, attention shifted to the ‘mystery of birth’, and the direct promise of the spectacle of childbirth became a frequent advertising tactic. For instance, publicity material for *The Birth of a Baby* (1938) proclaimed, ‘See a baby born before your very eyes!’^53^ Initially intended as a ‘nontheatrical’ medical training film, *The Birth of a Baby* was presented to general audiences under the auspices of the American Committee on Maternal Welfare, an umbrella group consisting of several reputable medical and public-health associations.^54^ It received generally positive reviews. *Time* praised the ‘absorbing example of visual education’ and *Variety* judged that it was ‘not in the class with so-called sex films’.^55^ Yet *The Birth of a Baby* went on to become one of the most controversial films of the 1930s: ‘The cinema explodes the stork myth’, proclaimed journalist Geraldine Sartain. ‘Almost overnight’, she claimed, ‘*The Birth of a Baby* became the most discussed picture since *The Birth of a Nation*’.^56^

**Figure 9.**
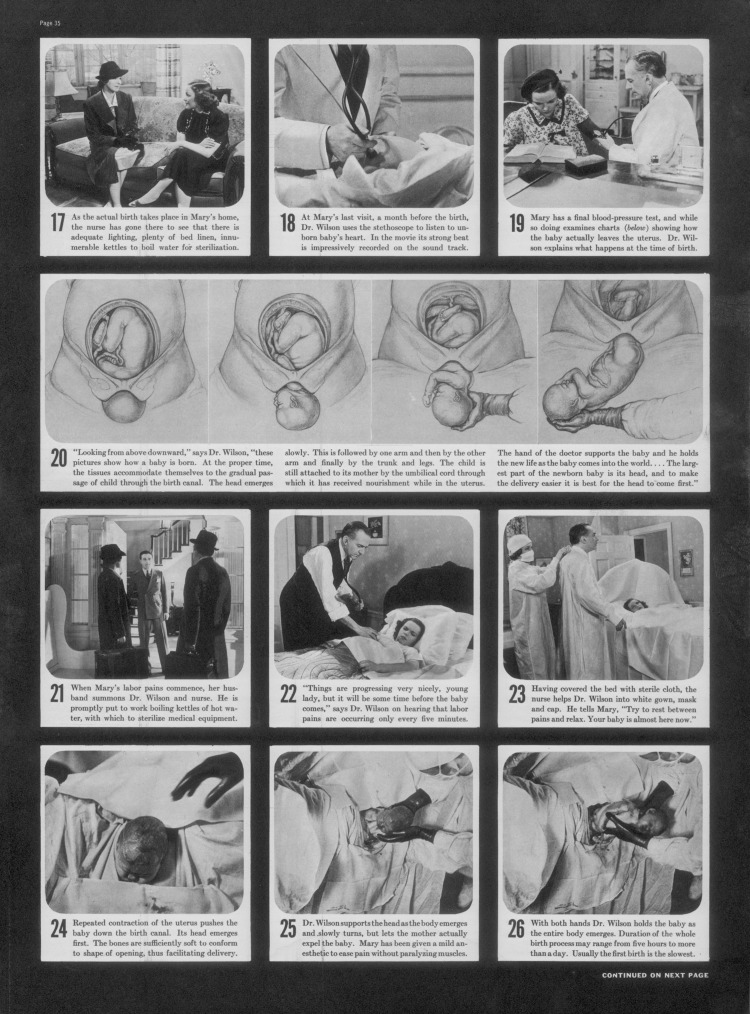
A page from the controversial ‘Birth of a baby’ photo-essay in *Life* magazine, 11 April 1938, p. 35, including three frames of the childbirth scene (bottom row); note the total covering in white cloth of the patient to preserve her modesty, a typical convention of such films. Pictures © Special Pictures Inc. Text used with permission of Time Inc.

A major debate over *The Birth of a Baby* was sparked not directly by the film itself, but indirectly by a photo-essay in *Life* magazine on 11 April 1938 (Figure 9). The magazine's publisher, Roy E. Larsen, sent warnings to subscribers, explaining that the section with the pictures could be removed, but in many cases the magazine arrived before his letter. The issue was banned in Canada as well as in several American states and cities; news dealers were arrested. Larsen had himself arrested for selling an ‘obscene’ magazine. He was ‘promptly acquitted’, as were most of the dealers. The issue sold out and the film did ‘tremendous business’.^57^ *The Birth of a Baby* played in cinemas for some twenty years and inspired imitators like *Childbirth from Life* (*c*.1938), *Life* (1938), *Childbirth* (1940) and *The Birth of a Child* (1938), the last of which provoked a ‘series of suits and countersuits’.^58^

The exploitation circuit was not the only route whereby obstetric films found non-medical audiences. In 1947, Austrian émigré Amos Vogel and his wife Marcia established Cinema 16 in New York City. Though better known today for cultivating the first American audience for European and home-grown experimental art films, the Vogels also programmed medical and scientific films, alongside documentaries, propaganda and other oddities. Cell division, sexual development and childbirth were consistently among their favourite themes.^59^ Reactions to childbirth were especially strong. For instance, one (female) commentator wrote of *Childbirth: Normal Delivery* (1950), screened in 1953 at Cinema 16, that the medical film depicted ‘the actual birth of a baby so graphically that the baby seems to leap simultaneously into the world and through the screen at the audience’.^60^ Cinema 16 eventually became a membership society to evade state censorship law, but before that Vogel ran into trouble with a documentary about not childbirth, but the birth of kittens.

In *The Private Life of a Cat* (1944), Alexander Hammid and Maya Deren, the first power couple of American avant-garde cinema, documented the arrival of their pet cat's kittens in their West Village apartment.^61^ Their cat Glamour Girl had previously ‘amazed’ the couple by calling them ‘to her side while she was delivering, unlike other cats who are known to prefer to hide away from people when they give birth’. This provided Hammid and Deren with the ‘key impetus to make the film’, which they narratively structured around the ‘act of delivery’.^62^ Hammid later described filming the birth scene:

To film her, it was necessary to only place her box more in the open, so I could get around with my camera. She seemed to mind little that her box was near the window – contrary to the belief that cats give birth only in dark places. The strong lights I needed turned on only for a few seconds necessary to take each shot. At first she disliked this, but when delivery got under way she was too busy to mind … almost none of the film is staged. Usually, I waited for the cats to do what I knew they would do from habit, or often I waited for a surprise, as in the case of the father seeing his children for the first time. My only contrivance was placing the kittens where I wanted them, making them look one way or the other by some noise, motion, or food. The film was taken over a period of four weeks … some seemingly consecutive shots were in reality taken days apart. Others, like the delivery, are seen in almost the same actual continuity.^63^

Vogel's programme notes praised the film, singling out the delivery scene: ‘Birth is shown as a tender, yet painful miracle, the very objectivity of portrayal robbing it of all sensationalism’.^64^ A positive review in the magazine *Popular Photography* similarly praised Hammid as a ‘sensitive and original thinker-with-the-camera’ and his film as ‘unusual’, ‘absorbing’, ‘charming’ and ‘instructive’. It did not remark on the birth scene one way or another, but did laud the ‘marvellous sequences in which the kittens take their first faltering steps, and lurch along as uncoordinated as any new-born baby’.^65^ The censors took a different view. As Vogel later lamented in his classic book, *Film as a Subversive Art* (1976), state censors banned the film in 1948 ‘as “indecent” because of its moving birth sequences’.^66^ Hammid later recalled the incident as ‘funny’.^67^

That even the birth of kittens was censored in the late 1940s provides some indication of the degree to which norms have changed since then. Human reproduction, however, is a somewhat different story. In 1957, BBC TV's flagship current affairs programme *Panorama* made headlines around the world when it broadcast footage of an ‘actual birth’ for the first time. In this issue Salim Al-Gailani recovers the history of the production, distribution and screening of the footage, originally intended for use in antenatal classes, in the context of heated debates over ‘natural childbirth’. Meanwhile, just as censorship was beginning to relax, experimental filmmakers found new ways of pushing boundaries, including by visually exploring the sexual and reproductive *human* body.^68^

## Between medicine and pornography

In contrast to the no-longer controversial birth of kittens, the spectacle of childbirth still has the power to shock. Take, for example, *Window Water Baby Moving* (1959), by pioneering experimental filmmaker Stan Brakhage. This intentionally silent, ambient study of his wife Jane's pregnancy and the birth of their first child Myrrena has become part of the canon of experimental film.^69^ Here it is worth noting, with medical film scholar Kirstin Ostherr, that it was the ‘graphic, bloody, close-up views of childbirth’ that caused trouble for the landmark film.^70^ It is also worth considering the highly contingent circumstances that led to its production. Brakhage had no intention of making a childbirth film when his pregnant wife Jane insisted on his presence during labour. When her doctor learned that the ‘expectant father’ was a filmmaker, he commissioned a movie that he would be able to show to his patients and their husbands. The camera, then, was to be Stan's passport to the delivery room – that is, until the hospital's management had second thoughts and retracted their initial offer to allow filming. The doctor, undeterred, agreed to a home delivery, an uncommon practice by the late 1950s. To make it happen, Brakhage, as he later recalled, was required to ‘hire a nurse and rent some very expensive emergency equipment’.^71^

Reactions were strong and began in pre-production, well before the film was even edited, much less projected for the first time. The Kodak laboratory where Brakhage had his film developed threatened to notify the police and only released his footage when Jane's doctor wrote a letter. Of the New York premiere in December 1959, film critic Jonas Mekas wrote for his column in the *Village Voice*, ‘On the screen probably for the first time ever in film history: a woman gives birth to a child. We see it all. The woman is ecstatic. And so is the father. The audience is totally totally silent’.^72^ After the screening, for which Stan was the projectionist, Maya Deren, his mentor and now the ‘grandmother’ of American avant-garde cinema, took the stage to declare emphatically that childbirth was a ‘private matter’ and should never be made public: ‘Even the animals, when they give birth, retreat into a secret place’, she proclaimed.^73^ Men reportedly ‘threw up’ and ‘rushed out’ of early screenings in ‘revulsion and panic’.^74^

**Figure 10.**
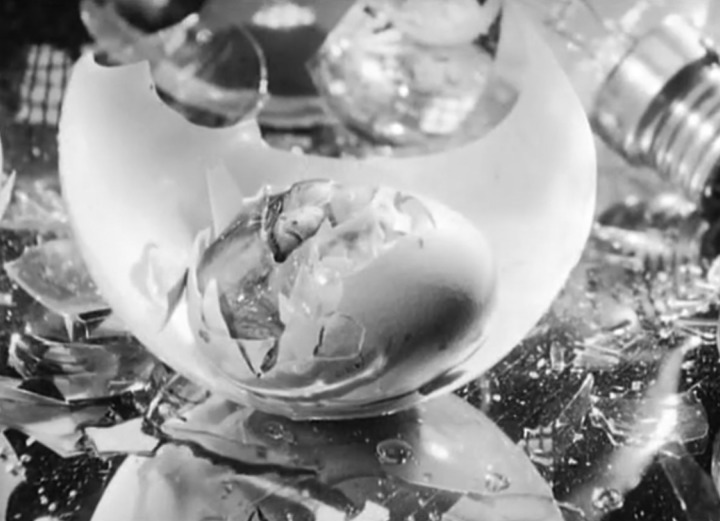
Screen capture from Agnès Varda's *L'opéra mouffe* (1958) showing a chick hatching in a shattered light bulb. The title refers to Rue Mouffetard, in Paris, where documentary elements of the experimental film play out.

Women, in turn, made their own, no less challenging, films about pregnancy and childbirth, notably Agnès Varda's *L'opéra mouffe* (1958), Gunvor Nelson's *Kirsa Nicholina* (1969) and Marjorie Keller's *Misconception* (1977).^75^ Varda, the ‘mother of French New Wave cinema’, described *L'opéra mouffe* as the ‘notebook of a pregnant woman unafraid to show what pregnancy really looks like’ (Figure 10).^76^ One critic contrasted Nelson's ‘open and matter-of-fact’ take on childbirth in *Kirsa Nicholina* with Brakhage's ‘melodramatic fascination’ with the pregnant and birthing body.^77^ And Keller, a student of Brakhage's, used the newly affordable synchronized sound recording technology of Super-8 explicitly to challenge what she saw as her teacher's silent ‘idealizations’;^78^ *Misconception*, as she soon after explained in an interview, was her ‘loving critique’ of *Window Water Baby Moving*.^79^

Performance artist and filmmaker Carolee Schneemann responded somewhat differently to Brakhage's provocation. She worried that Brakhage's ‘male eye replicated or possessed the vagina's primacy of giving birth’. Moreover, she ‘wanted to see “the fuck,” lovemaking's erotic blinding core apart from maternity/paternity’. The result was Schneemann's own sexually explicit *Fuses* (1967), a still more controversial landmark in the history of experimental film.^80^ Yet, even as Schneemann critiqued *Window Water Baby Moving*, she acknowledged Brakhage's ‘unique … willingness to focus on the actual birth’. ‘You must understand’, she later recalled, ‘there were no precedents that we know of – only medical and pornographic models’.^81^

Considering that *Window Water Baby Moving* was surely not the medical training film that Jane's doctor had bargained for, it is all the more remarkable that thousands of 8 mm prints circulated in maternity clinics in the 1960s, where it often screened on a double bill with George Stoney's *All My Babies* (1952), a lyrical documentary about midwifery in the American South.^82^ But Brakhage's film lived on mainly as a staple of film studies syllabuses.^83^ ‘That's a film I cannot teach without’, remarked a film scholar in the late 1990s. ‘I show it in nearly every course’, he continued; ‘many of my students are amazed, shocked; they swear off being parents! Anyway, forty years later it's still one of the most powerful films I can show’.^84^ Today, *Window Water Baby Moving* remains a ‘rite of initiation’ for students, who, as another film scholar recently remarked, ‘tend to respond to it as either the “most beautiful” or the “most obscene” film they have ever seen’.^85^ The film's undiminished visual impact speaks to the dearth of non-sanitized, realistic portrayals of childbirth, even in the present context of a mass culture persistently saturated with reproduction.^86^

It is worth dwelling on a final, little-remarked aspect of *Window Water Baby Moving*. As Brakhage later recalled,

Jane had German measles at three months, and in those days, they thought this meant there was a much higher chance of giving birth to a monster. We were always concerned about that. When I first looked through the camera at the baby emerging, I thought, ‘This *is* a monster!’ I had never seen a newborn baby before and thought this was a deformed monster, and I remember the thought passing through my head, ‘Then I will make a monster film!’ and continued to film in a kind of rage. Of course, it turned out much happier than that.^87^

Several features of this eye-opening anecdote merit discussion. First, the disease. The couple's concerns were not in fact unfounded. As was already suspected in the 1950s, contracting rubella (German measles) in pregnancy does increase the risk of giving birth to a malformed child and highly mediatized epidemics of the disease would soon after play a significant role in the liberalization of abortion law in the United States and other countries.^88^ Second, Brakhage had ‘never seen a newborn baby’ until filming the delivery of his first child; birth imagery was scarce in the 1950s and fathers were still banned from the delivery room.^89^ Third, the ‘monster movie’. Though Brakhage may have been thinking of ‘classic’ 1930s horror films such as *Frankenstein* (1931) or *Island of Lost Souls* (1932), the Cold War reinvigorated the genre, which traded old fears for new ones about radioactive fallout, mutation and degeneration.^90^ Between the medical reality of rubella and the cinematic fantasy of nuclear disaster, it is perhaps understandable that Brakhage, an avid filmgoer and naive father-to-be, momentarily feared he was casting his daughter in a monster movie.^91^

## From taboo to cliché

As with graphic nudity, sex and other taboos, reproduction on screen has become something of a cliché: part of a broader liberalizing trend across a wide range of mainstream media that really got going in the 1960s and 1970s.^92^ From *A Taste of Honey* (1961) to *Up the Junction* (1968), British ‘kitchen sink’ cinema looked to recent novels and stage plays for gritty narratives about unmarried pregnancy and abortion; Ken Loach's *Poor Cow* (1967) opened with documentary footage of childbirth.^93^ New reproductive technologies supplied plotlines, as with *Prudence and the Pill* (1968), a comedy film based on a novel and folk tale about a daughter taking her mother's contraceptive pills and replacing them with aspirin, resulting in the mother becoming pregnant.^94^

**Figure 11.**
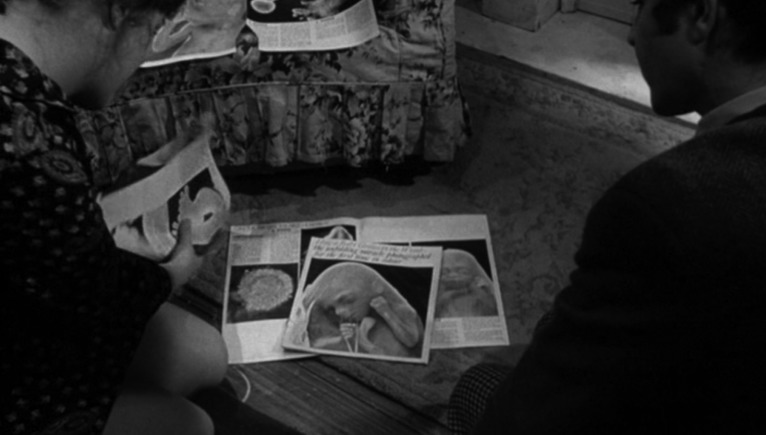
Screen capture from Silvio Narizzano's 1966 adaptation of Margaret Foster's *Georgy Girl* (1965) showing flatmates Georgina Parkin (Lynn Redgrave) and Jos Jones (Alan Bates) marvelling at Nilsson's photographs republished in the *Sunday Times* magazine. Georgina refers to them as ‘the most marvellous pictures’, but for Jos's pregnant girlfriend Meredith (Charlotte Rampling), not pictured here, they are a ‘chamber of horrors’. Produced and distributed by Columbia Pictures.

The public visibility of human embryos increased when colour pictures appeared on 30 April 1965 on the cover of *Life* magazine and then in Swedish photographer Lennart Nilsson's global bestseller, *A Child Is Born*.^95^ The flatmate and boyfriend of a pregnant woman are together seen marvelling at Nilsson's photographs in *Georgy Girl* (1966), a British film that also features the same pair watching childbirth on television and reading Dr Benjamin Spock's leading pregnancy advice manual, *Baby and Child Care* (Figure 11). The photographs soon provided the model for the ‘star child’, a foetus floating in space and the next step in human evolution in Stanley Kubrick's *2001: A Space Odyssey* (1967).^96^ The classic ‘repro-horror’ films, *Rosemary's Baby* (1968) and *Alien* (1979), bookended a decade obsessed with the unsettled and increasingly contested contents of the womb.^97^ Activists in the 1970s both made and protested films about reproductive rights, transforming movie theatres and film festivals into sites of occasionally violent confrontation.^98^

In the 1980s, after several Western countries had legalized abortion and the first ‘test-tube babies’ had been born, ‘pro-life’ activists mobilized *in utero* videography, most notoriously in *The Silent Scream* (1984), while infertility experts made television documentaries about the ‘miracle’ of IVF.^99^ ‘Body horror’ films such as *The Fly* (1986) incorporated pregnancy, miscarriage and abortion into storylines about genetic contamination and monstrous hybridity.^100^ The announcement in 1997 of the birth of Dolly the cloned sheep shifted cinematic narratives about human cloning ‘from horror to hope’,^101^ while ‘repro-dystopian’ films, from *The Handmaid's Tale* (1990) to *Children of Men* (2006), continued to evoke abiding concerns about reproductive control.^102^ Today, the *Alien* franchise is still going strong, with the most recent instalment, *Alien: Covenant* (2017), continuing to engage in a sophisticated way with reproduction, from embryo to fully grown monster. *The Handmaid's Tale* lives again as a critically acclaimed television series, provoking fresh discussion on the enduring relevance of its source material, the 1985 dystopian novel by Canadian author Margaret Atwood.

So much has changed since Lucille Ball made prime-time history in 1952 as the first pregnant actress to play a pregnant character, on the popular American comedy series *I Love Lucy*.^103^ From *The Kids Are All Right* (2010), an independent film about a married lesbian couple who meet the sperm-donor father of their children, to *Two 4 One* (2014), a crowdfunded film about a transgender man who accidentally becomes pregnant, unconventional family forms have inspired new takes on older themes, queering reproduction on film.^104^ Changing norms have in some cases been bolstered by advertising campaigns. As Jesse Olszynko-Gryn shows in this issue, product placement helped from the 1990s to propel the commercial rise of the Clearblue brand of home pregnancy test by making it a fixture of boundary-pushing British soap operas that increasingly looked to unmarried teenage pregnancy and abortion for dramatic plotlines.

Depictions of pregnancy and childbirth on television have become a commonplace, including on so-called reality programmes such as MTV's *16 and Pregnant* and Channel 4's *One Born Every Minute*.^105^ Six of the more than 250 children born on *One Born Every Minute* were recently filmed watching their televised births for the spin-off miniseries *I Was Born on One Born*. As recently as the 1980s, in contrast, home movies were still a novelty and the birth of a child could stimulate parents to purchase their first Polaroid instant-film camera or video camcorder.^106^ Today's ubiquitous smartphones are also digital video cameras and, since the inception of YouTube in 2005, the Internet has increasingly played host to homemade childbirth videos, some of which have stirred considerable controversy.^107^

Companies, meanwhile, offer ‘keepsake’ ultrasound scans, displayed on large LCD screens, and recorded to DVD, ‘usually with a soundtrack of the client's choice’.^108^ It is now possible to watch, in the comfort of one's home or on one's phone, pioneering embryological films, vintage sex education films, or the latest computer simulations of zebra fish development. Reproductive scientists and clinicians routinely generate, display and analyse moving images, including of human conception in a Petri dish.^109^ Journal articles and textbooks continue to matter in important ways, but embedded or linked videos play an increasingly prominent role.^110^ Teachers rely on movies to broach sensitive subjects and broaden classroom discussion, including about reproduction.^111^ Feminist documentaries have stirred debate on everything from painless, even ‘orgasmic’, childbirth to India's booming surrogacy industry.^112^

*Reproduction on Film* began as series of public film screenings and discussions, activities that have fed back into the research process in ways that film appears to facilitate most effectively. This introduction has explored some of the media-specific hopes and fears associated with the history of reproduction in cinema, and on television and video: the potent realism of the moving image, its fraught visual politics, and the disruptive power of innovative distribution networks – from roadshows to the Internet – to collapse boundaries between genres. Focusing on one neglected medium always throws taken-for-granted features of other media into relief, and reproduction, as a theme, seems to have an intensifying effect. It opens up resonant issues, from the manipulation of scale, time and motion in research and teaching to the contested visual communication of biological and medical subjects to laypeople. From postcards to time-lapse, cabbage fairies to IVF, communication technologies and reproductive technologies have structured the stories we tell about making or not making babies. The rest of this special issue will set the stage for further exploration of a vast and still mostly uncharted history.

## Footnotes

1 For a recent exploration of reproduction as it relates to communication technologies see Nick Hopwood, Peter Murray Jones, Lauren Kassell and Jim Secord (eds.), Communicating Reproduction, a special issue of the *Bulletin of the History of Medicine* (2015) 89, pp. 379–55610.1353/bhm.2015.0064PMC465558226521666. For a broader survey of the field see Nick Hopwood, Rebecca Flemming and Lauren Kassell (eds.), Reproduction: Antiquity to the Present Day, Cambridge: Cambridge University Press, forthcoming 2018.

2 Julien Duvivier and Henry Lepage's French film was first released in 1924, and rereleased as an updated version in 1933. In French, the title is *La machine à refaire la vie*; the standard translation in English has *refaire* as ‘re-create’.

3 On genetics and eugenics in film see Martin Pernick, The Black Stork: Eugenics and the Death of ‘Defective’ Babies in American Medicine and Motion Pictures since 1915, Oxford: Oxford University Press, 1996; David A. Kirby, ‘The new eugenics in cinema: genetic determinism and gene therapy in *GATTACA*’, Science Fiction Studies (2000) 27, pp. 193–215; Kirby, ‘The devil in our DNA: a brief history of eugenics in science fiction films’, Literature and Medicine (2007) 26, pp. 83–1081829036310.1353/lm.2008.0006; Jackie Stacey, The Cinematic Life of the Gene, Durham, NC: Duke University Press, 2010; and Angela M. Smith, Hideous Progeny: Disability, Eugenics, and Classic Horror Cinema, New York: Columbia University Press, 2011. On sex education in film see Robert Eberwein, Sex Ed: Film, Video, and the Framework of Desire, New Brunswick, NJ: Rutgers University Press, 1999; Uta Schwarz, ‘*Helga* (1967): West German sex education and the cinema in the 1960s’, in Lutz D.H. Sauerteig and Roger Davidson (eds.), Shaping Sexual Knowledge: A Cultural History of Sex Education in Twentieth-Century Europe, London: Routledge, 2009, pp. 197–213; Elisabet Björklund, ‘The most delicate subject: a history of sex education films in Sweden’, PhD thesis, Lund University, 2012; Manon Parry, Broadcasting Birth Control: Mass Media and Family Planning, New Brunswick, NJ: Rutgers University Press, 2013; Anita Winker, ‘Biology, morality and gender: East and West German sex education in films, 1945–70’, PhD thesis, Durham, 2014; and Christian Bonah and Anja Laukötter (eds.), Screening Diseases: Films on Sex Hygiene in Germany and France in the First Half of the 20th Century, a special issue of *Gesnerus* (2015) 72, pp. 5–9326403052.

4 See, for example, Meredith Nash, Making ‘Postmodern’ Mothers: Pregnant Embodiment, Baby Bumps and Body Image, New York: Palgrave Macmillan, 2012; and Joyce Peterson, ‘Baby M: American feminists respond to a controversial case’, Journal of Women's History (2016) 28, pp. 103–125.

5 See Tania Modleski, ‘Three men and Baby M’, Camera Obscura (1986) 6, pp. 68–81; Mary Desjardins, ‘*Baby Boom*: the comedy of surrogacy in film and television’, Velvet Light Trap (1992) 29, pp. 21–30; and E. Ann Kaplan, ‘Look who's talking, indeed: fetal images in recent North American visual culture’, in Evelyn Nakano Glenn, Grace Chang and Linda Rennie Forcey (eds.), Mothering: Ideology, Experience, and Agency, New York: Routledge, 1994, pp. 121–137.

6 Rosalind Pollack Petchesky, ‘Fetal images: the power of visual culture in the politics of reproduction’, Feminist Studies (1987) 13, pp. 263–292; and Jennifer Taylor, ‘The public foetus and the family car: from abortion politics to a Volvo advertisement’, Public Culture (1992) 4, pp. 67–80.

7 See Parenting and Reproduction, a special issue of *Velvet Light Trap* (1992) 29, pp. 1–65; E. Ann Kaplan, Motherhood and Representation: The Mother in Popular Culture and Melodrama, London: Routledge, 1992; Donna Bassin, Margaret Honey and Meryle Mahrer Kaplan (eds.), Representations of Motherhood, New Haven, CT: Yale University Press, 1994; and Lucy Fischer, Cinematernity: Film, Motherhood, Genre, Princeton, NJ: Princeton University Press, 1996. A somewhat earlier influential example is Linda Williams, ‘“Something else besides a mother”: *Stella Dallas* and the maternal melodrama’, Cinema Journal (1984) 24, pp. 2–27.

8 See Heather Addison, Mary Kate Goodwin-Kelly and Elaine Roth (eds.), Motherhood Misconceived: Representing the Maternal in U.S. Films, Albany: State University of New York Press, 2009; Tanja Nusser, ‘Wie sonst das Zeugen Mode war’: Reproduktionstechnologien in Literatur und Film, Berlin: Rombach, 2011; Kelly Oliver, Knock Me Up, Knock Me Down: Images of Pregnancy in Hollywood Film, New York: Columbia University Press, 2012; Parley Ann Boswell, Pregnancy in Literature and Film, Jefferson, NC: McFarland, 2014; and Claire Jenkins, Home Movies: The American Family in Contemporary Hollywood Cinema, London: I.B. Tauris, 2015.

9 On the history of film, models and images as visual technologies of science communication see Soraya de Chadarevian and Nick Hopwood (eds.), , Stanford, CA: Stanford University Press, 2004; Luc Pauwels (ed.), , Hanover, NH: University Press of New England, 2006; Timothy Boon, Films of Fact: A History of Science in Documentary Films and Television, London: Wallflower, 2008; David A. Kirby, Lab Coats in Hollywood: Science, Scientists, and Cinema, Cambridge, MA: MIT Press, 2011; Scott Curtis, The Shape of Spectatorship: Art, Science, and Early Cinema in Germany, New York: Columbia University Press, 2015; Oliver Gaycken, Devices of Curiosity: Early Cinema and Popular Science, New York: Oxford University Press, 2015; Nick Hopwood, Haeckel's Embryos: Images, Evolution, and Fraud, Chicago: The University of Chicago Press, 2015; and Michael Sappol, Body Modern: Fritz Kahn, Scientific Illustration, and the Homuncular Subject, Minneapolis: University of Minnesota Press, 2017.

10 See Virgilio Tosi, Cinema before Cinema: The Origins of Scientific Cinematography, London: British Universities Film and Video Council, 2005.

11 For postcards as they relate to early film see Lauren Rabinovitz, Electric Dreamland: Amusement Parks, Movies, and American Modernity, New York: Columbia University Press, 2012, pp. 96–134.

12 See Jacques Véron and Jean-Marc Rohrbasser, Bébés, familles et cartes postales de 1900 à 1950, Paris: Ined, 2015; for an analysis of the German tradition see Christina Benninghaus, ‘“No, thank you, Mr Stork!”: voluntary childlessness in Weimar and contemporary Germany’, Studies in the Maternal (2014) 6, pp. 1–36, available at http://doi.org/10.16995/sim.8.

13 For postcard landscapes and scenes as they relate to film see Jennifer Lynn Peterson, Education in the School of Dreams: Travelogues and Early Nonfiction Film, Durham, NC: Duke University Press, 2013.

14 Jane M. Gaines, ‘Of cabbages and authors’, in Jennifer M. Bean and Diane Negra (eds.), , Durham, NC: Duke University Press, 2002, pp. 88–118; and Gaines, ‘First fictions’, Signs (2004) 30, pp. 1293–1317.

15 See Alison McMahan, Alice Guy Blaché: Lost Visonary of the Cinema, New York: Bloomsbury Academic, 2014, p. 39. For essays on Guy and other women pioneers of early cinema see Jane Gaines, Radha Vatsal and Monica Dall'Asta (eds.), , Center for Digital Research and Scholarship, New York: Columbia University Libraries, 2013, available at https://wfpp.cdrs.columbia.edu.

16 See, for example, Marta Braun, Charles Keil, Rob King, Paul Moore and Louis Pelletier (eds.), , New Barnet: John Libbey, 2016. For a recent review of early cinema as it relates to science see Jesse Olszynko-Gryn, ‘Film lessons: early cinema for historians of science’, BJHS (2016) 49, pp. 279–2862735394610.1017/S000708741600039X.

17 See Tom Gunning, ‘“Primitive” cinema: a frame-up? Or the trick's on us’, Cinema Journal (1989) 28, pp. 3–12; and Scott Higgins, ‘The silent screen, 1895–1927: editing’, in Charlie Keil and Kristen Whissel (eds.), , New Brunswick, NJ: Rutgers University Press, 2016, pp. 22–36.

18 The film *Artistic Creation* and dozens more as well as ‘Robert Paul: time traveller’, a historical essay by Ian Christie, are included with the DVD *RW Paul: The Collected Films, 1895–1980*, London: BFI, 2006. See also Ian Christie, ‘The visible and the invisible: from “tricks” to “effects”’, (2015) 13, pp. 105–112; and Malcolm Cook, ‘The lightning cartoon: animation from music hall to cinema’, Early Popular Visual Culture (2013) 11, pp. 237–254.

19 Pernick, op. cit. (3), p. 51; Laura Doan, ‘Sex education and the Great War soldier: a queer analysis of the practice of “hetero” sex’, Journal of British Studies (2012) 51, pp. 641–663. *Whatsoever a Man Soweth* is available on DVD in the landmark collection of British sex education films, *The Birds and the Bees*, London: BFI, 2009 (originally titled *The Joy of Sex Education*).

20 See Andrew Shail (ed.), , a special issue of *Early Popular Visual Culture* (2013) 2, pp. 97–177.

21 Pernick, op. cit. (3), p. 123.

22 See Pernick, op. cit. (3), p. 130. On Porter, who made films for the prolific Edison company, see Charles Musser, Before the Nickelodeon: Edwin S. Porter and the Edison Manufacturing Company, Berkeley: University of California Press, 1991.

23 On incubator shows see William A. Silverman, ‘Incubator-baby side shows’, Pediatrics (1979) 2, pp. 127–141382078; Pernick, op. cit. (3), pp. 54, 112. On hydrotherapy see George Weisz, ‘Spas, mineral waters, and hydrological science in twentieth-century France’, Isis (2001) 93, pp. 451–48310.1086/38527811810892.

24 See Jeffrey A. Dym, Benshi, Japanese Silent Film Narrators, and Their Forgotten Narrative Art of Setsumei: A History of Japanese Silent Film Narration, Lewiston, NY: Edwin Mellen Press, 2003.

25 See Julian Graffy, Bed and Sofa, London: I.B. Tauris, 2001. On abortion in Soviet Russia see Wendy Z. Goldman, ‘Women, abortion and the state, 1917–36’, in Barbara Evans Clements, Barbara Alpern Engel and Christine D. Worobec (eds.), Russia's Women: Accommodation, Resistance, Transformation, Berkeley: University of California Press, 1991, pp. 243–266. On the history of abortion in film and television see Andrea L. Press and Elizabeth R. Cole, Speaking of Abortion: Television and Authority in the Lives of Women, Chicago: The University of Chicago Press, 1999; Ursula von Keitz, Im Schatten des Gesetzes: Schwangerschaftskonflikt und Reproduktion im deutschsprachigen Film 1918 bis 1933, Marburg: Schüren, 2005; Heather MacGibbon, ‘The abortion narrative in American film: 1900–2000’, PhD thesis, New York University, 2007; Fran Bigman, ‘“Nature's a wily dame”: abortion in British literature and film, 1907–1967’, PhD thesis, University of Cambridge, 2014; Megan Lynn Minarich, ‘Hollywood's reproduction code: regulating contraception and abortion in American cinema, 1915–1952’, PhD thesis, Vanderbilt University, Nashville, Tennessee, 2014; Gretchen Sisson and Katrina Kimport, ‘Telling stories about abortion: abortion-related plots in American film and television, 1916–2013’, Contraception (2014) 89, pp. 413–4182451293810.1016/j.contraception.2013.12.015; and Chika Kinoshita, ‘Something more than a seduction story: Shiga Akiko's abortion scandal and late 1930s Japanese film culture’, Feminist Media Histories (2015) 1, pp. 29–63.

26 Graham Roberts, The Man with the Movie Camera, London: I.B. Tauris, 2000, p. 70.

27 Sergei Eisenstein, Disney, Berlin: Potemkin Press, 2013.

28 See Anja Laukötter, ‘Listen and watch: the practice of lecturing and the epistemological status of sex education films in Germany’, Gesnerus (2015) 72, pp. 56–7626403055.

29 Quoted in Anne Nesbet, Savage Junctures: Sergei Eisenstein and the Shape of Thinking, London: I.B. Tauris, 2003, p. 140.

30 Nesbet, op. cit. (29), p. 142.

31 Eisenstein, op. cit. (27), p. 117. In Ernst Haeckel's then widely discussed theory of evolution, initially formless embryos climbed the ‘ladder of evolution’ in the womb. See Hopwood, op. cit. (9).

32 See, for example, Karen Beckmann (ed.), Animating Film Theory, Durham, NC: Duke University Press, 2014.

33 Anthony R. Michaelis, Research Films in Biology, Anthropology, Psychology, and Medicine, New York: Academic Press, 1955, p. 85. For the classic critique of the ‘male’ sperm as active and the ‘female’ egg as passive see Emily Martin, ‘The egg and the sperm: how science has constructed a romance based on stereotypical male–female roles’, Signs (1991) 16, pp. 485–501.

34 Michaelis, op. cit. (33), p. 117. For time-lapse as it relates to botanical research and Darwinism see Oliver Gaycken, ‘The secret life of plants: visualizing vegetative movement, 1880–1903’, Early Popular Visual Culture (2012) 10, pp. 51–69; Gaycken, ‘Early cinema and evolution’, in Bernhard V. Lightman and Bennett Zon (eds.), Evolution in Victorian Culture, Cambridge: Cambridge University Press, 2014, pp. 94–120.

35 Nick Hopwood, ‘Visual standards and disciplinary change: normal plates, tables and stages in embryology’, History of Science (2005) 43, pp. 239–303. See also the online resource: Tatjana Buklijas and Nick Hopwood, *Making Visible Embryos* (2008–2010; last reviewed 2014), at www.hps.cam.ac.uk/visibleembryos.

36 See Marta Braun, Picturing Time: The Work of Etienne-Jules Marey (1830–1904), Chicago: The University of Chicago Press, 1992; Philip Prodger, Time Stands Still: Muybridge and the Instantaneous Photography Movement, New York: Oxford University Press, 2003; Rebecca Solnit, River of Shadows: Eadweard Muybridge and the Technological Wild West, New York: Viking, 2003; and Marta Braun, Eadweard Muybridge, London: Reaktion Books, 2010.

37 See Hannah Landecker, ‘The life of movement: from microcinematography to live-cell imaging’, Journal of Visual Culture (2012) 11, pp. 378–399; and Oliver Gaycken, ‘“A living, developing egg is present before you”: animation, scientific visualization, and modeling’, in Beckmann, op. cit. (32), pp. 68–81.

38 Ries quoted in translation in Christopher Kelty and Hannah Landecker, ‘A theory of animation: cells, L-systems, and film’, Grey Room (2004) 17, pp. 30–63, 37.

39 See Hannah Landecker, ‘The Lewis films: tissue culture and “living anatomy,” 1919–1940’, in Jane Maienschein, Marie Glitz and Garland E. Allen (eds.), Centennial History of the Carnegie Institution of Washington, vol. 5: *The Department of Embryology*, Cambridge: Cambridge University Press, 2004, pp. 117–144; and Kirsten Ostherr, ‘Animating informatics: scientific discovery through documentary film’, in Alexandra Jahusz and Alisa Lebow (eds.), A Companion to Contemporary Documentary Film, New York: John Wiley & Sons, 2015, pp. 280–297.

40 Michaelis, op. cit. (33), pp. 115–117.

41 Michaelis, op. cit. (33), p. 117.

42 Lynn M. Morgan, Icons of Life: A Cultural History of Human Embryos, Berkeley: University of California Press, 2009, p. 200. See further Emily K. Wilson, ‘Ex utero: live human fetal research and the films of Davenport Hooker’, Bulletin of the History of Medicine (2014) 88, pp. 132–1602476980510.1353/bhm.2014.0002. Hooker's 1952 film is available via the Wellcome Library's ‘reproduction’ playlist at www.youtube.com/user/WellcomeFilm/playlists.

43 Maverick surgeon Eugene Louis Doyen began filming in the operating theatre as early as 1898. See Thierry Lefebvre, *La chair et le celluloïd: Le cinéma chirurgical du docteur Doyen*, Paris: J. Doyen, 2004.

44 ‘Foreign letters: Paris’, Journal of the American Medical Association (December 1921) 77(24), pp. 2071–2072.

45 Our discussion builds on the analysis of wax models as they relate to print media and pedagogy in Nick Hopwood, ‘Plastic publishing in embryology’, in de Chadarevian and Hopwood, op. cit. (9), pp. 170–206. See further Hopwood, Embryos in Wax: Models from the Ziegler Studio, with a Reprint of ‘Embryological Wax Models’ by Friedrich Ziegler, Cambridge: Whipple Museum of the History of Science, 2012.

46 See Devin Orgeron, Marsha Orgeron and Dan Streible (eds.), Learning with the Lights Off: Educational Film in the United States, Oxford: Oxford University Press, 2012; and Haidee Wasson, ‘Electric homes! Automatic movies! Efficient entertainment! 16 mm and cinema's domestication in the 1920s’, Cinema Journal (2009) 48, pp. 1–21.

47 New York University Film Library, A Catalogue of Selected 16 mm. Educational Motion Pictures, New York: NYU, 1950, pp. 80–81.

48 NYUFL, op. cit. (47), p. 139.

49 On British film censors and contraception see Annette Kuhn, ‘The “Married Love” affair’, Screen (1986) 27, pp. 5–21; Kuhn, Cinema, Censorship and Sexuality, 1909–1925, New York: Routledge, 1988; and Jessica Borge, ‘Propagating progress and circumventing harm: reconciling references to contraceptives in British television and cinema of the 1960s’, in Waltraud Maierhofer and Beth Widmaier Capo (eds.), Reproductive Rights Issues in Popular Media: International Perspectives, Jefferson, NC: McFarland, 2017, pp. 11–28.

50 Eric Schaefer, “Bold! Daring! Shocking! True!”: A History of Exploitation Films, 1919–1959, Durham, NC: Duke University Press, 1999, p. 8. On stag films and pornography see Linda Williams, Hard Core: Power, Pleasure and ‘Frenzy of the Visible’, London: Pandora, 1990.

51 Schaefer, op. cit. (50), pp. 2–5.

52 Schaefer, op. cit. (50), p. 6.

53 Schaefer, op. cit. (50), pp. 106–107.

54 Schaefer, op. cit. (50), p. 188.

55 Schaefer, op. cit. (50), p. 190.

56 Geraldine Sartain, ‘The cinema explodes the stork myth’, Journal of Educational Sociology (1938) 12, pp. 142–146, 144. *The Birth of a Nation* (1915), a controversial landmark of American cinema, dramatized the origins of the KKK. See Melvyn Stokes, D.W. Griffith's The Birth of a Nation*: A History of ‘The Most Controversial Motion Picture of All Time’*, Oxford: Oxford University Press, 2008.

57 Schaefer, op. cit. (50), p. 190. See also Benjamin Strassfeld, ‘A difficult delivery: debating the function of the screen and educational cinema through *The Birth of a Baby* (1938)’, Velvet Light Trap (2013) 72, pp. 44–57.

58 Schaefer, op. cit. (50), p. 191.

59 Kirsten Ostherr, Medical Visions: Producing the Patient through Film, Television, and Imaging Technologies, Oxford: Oxford University Press, 2013, p. 117.

60 Flora Rheta Schreiber, ‘New York: a cinema capital’, Quarterly of Film, Radio and Television (1953) 7, pp. 264–273, 266.

61 On Hammid, Deren and their cat film see Jaroslav Anděl, Alexandr Hackenschmied, Prague: Torst, 2000; John David Rhodes, Meshes of the Afternoon, London: BFI and Palgrave, 2011; and Rosalind Galt, ‘Cats and the moving image’, in Michael Lawrence and Laura McMahon (eds.), Animal Life and the Moving Image, London: BFI and Palgrave, 2015, pp. 42–57.

62 Michael Omasta and Alexander Hammid, ‘“The rest is more or less routine stuff”: Michael Omasta in correspondence with Alexander Hammid, Vienna/New York (Sept. 2001–Jan. 2002)’, in Michael Omasta (ed.), Tribute to Sasha: Das filmische Werk von Alexander Hammid, Vienna: Synema, 2002, pp. 157–176, 171.

63 Amos Vogel, ‘Program notes (Cinema 16, September 1948)’, in Omasta, op. cit. (62), p. 209.

64 Vogel, op. cit. (63), p. 209.

65 ‘The private life of a cat’, *Popular Photography* (April 1947) 20, p. 130.

66 Amos Vogel, *Film as a Subversive Art*, London: Weidenfeld and Nicolson, 1976, p. 262.

67 Omasta and Hammid, op. cit. (62), p. 171.

68 See Ara Osterweil, Flesh Cinema: The Corporeal Turn in American Avant-Garde Film, Manchester: Manchester University Press, 2014.

69 Brakhage's second of five childbirth films, *Thigh Line Lyre Triangular* (1961), was much more experimental and abstract. See William R. Barr, ‘Brakhage: artistic development in two childbirth films’, Film Quarterly (1976) 29, pp. 30–34; and Shira Segal, ‘Home movies and home birth: the avant-garde childbirth film and pregnancy in new media’, PhD thesis, Indiana University, 2011.

70 Ostherr, op. cit. (59), p. 120.

71 Ostherr, op. cit. (59), p. 124.

72 Jonas Mekas, ‘Recollections of Stan Brakhage’, in David E. James (ed.), Stan Brakhage: Filmmaker, Philadelphia, PA: Temple University Press, 2005, pp. 107–112, 107. The review was not, in fact, published at the time.

73 Mekas, op. cit. (72), p.107.

74 Carolee Schneemann, ‘It is painting’, in James, op. cit. (72), pp. 78–87, 83.

75 See Vogel, op. cit. (66), pp. 258–262; Robin Blaetz (ed.), *Women's Experimental Cinema: Critical Frameworks*, Durham, NC: Duke University Press, 2007; and Segal, op. cit. (69).

76 Mireille Amiel, ‘Agnès Varda talks about cinema’, in T. Jefferson Kline (ed.), Agnès Varda: Interviews, Jackson: University Press of Mississippi, 2014, pp. 64–77, 74.

77 Scott MacDonald, A Critical Cinema 3: Interviews with Independent Filmmakers, Berkeley: University of California Press, 1998, p. 182.

78 J. Hoberman, ‘The Super-80s’, Film Comment (1981) 17, pp. 39–43, 42. On sound recordings of childbirth see Paula Michaels, ‘The sounds and sights of natural childbirth: films and records in antenatal preparation classes, 1950s–1980s’, Social History of Medicine (2017)10.1093/shm/hkw119PMC591436429713123, Advance Access, doi: 10.1093/shm/hkw119.

79 Amy Taubin, ‘Discussion between Marjorie Keller and Amy Taubin’, Idiolects (1978) 6, pp. 28–31, 28; quoted in Roxanne Samer, ‘Re-conceiving *Misconception*: birth as a site of filmic experimentation’, Jump Cut (Summer 2011) 53, at www.ejumpcut.org/archive/jc53.2011/samerMisconception/text.html.

80 Schneemann, op. cit. (74), p. 83.

81 Interview with Kate Haug in Wide Angle (1977) 20, pp. 20–49, 23; republished in Carolee Schneemann, Imaging Her Erotics: Essays, Interviews, Projects, Cambridge, MA: MIT Press, 2002.

82 Ostherr, op. cit. (59), p. 125; and Lynne Jackson, ‘The production of George Stoney's film *All My Babies: A Midwife's Own Story* (1952)’, Film History (1987) 1, pp. 367–39212879506.

83 On the history of film studies see Michael Zryd, ‘Experimental film and the development of film study in America’, in Lee Grieveson and Haidee Wasson (eds.), Inventing Film Studies, Durham, NC: Duke University Press, 2008.

84 Scott MacDonald (ed.), A Critical Cinema 4: Interviews with Independent Filmmakers, Berkeley: University of California Press, 2005, pp. 62–63.

85 Osterweil, op. cit. (68), pp. 101–102.

86 See Imogen Tyler and Jessica Clements, ‘The taboo aesthetics of the birth scene’, Feminist Review (2009) 93, pp. 134–137.

87 MacDonald, op. cit. (84), p. 68, original emphasis. For Jane's perspective see Jane Brakhage, ‘The birth film’, in P. Adams Sitney (ed.), Film Culture Reader, New York: Praeger, 1970, pp. 230–233.

88 See Leslie Reagan, Dangerous Pregnancies: Mothers, Disabilities, and Abortion in Modern America, Berkeley: University of California Press, 2010; and Clare Parker, ‘From immorality to public health: thalidomide and the debate for legal abortion in Australia’, Social History of Medicine (2012) 25, pp. 863–880.

89 See Richard K. Reed, Birthing Fathers: The Transformation of Men in American Rites of Birth, New Brunswick, NJ: Rutgers University Press, 2005; and Judith Walzer Leavitt, Make Room for Daddy: The Journey from Waiting Room to Birthing Room, Chapel Hill: University of North Carolina Press, 2009. For the British history see Laura King, ‘Hiding in the pub to cutting the cord? Men's presence at childbirth in Britain *c*.1940s–2000’, Social History of Medicine (2017) 30, pp. 389–4072971311610.1093/shm/hkw057PMC5914339.

90 For example, Jerome F. Shapiro, Atomic Bomb Cinema: The Apocalyptic Imagination on Film, London: Routledge, 2002; on ‘monster movies’ see Andrew Tudor, Monsters and Mad Scientists: A Cultural History of the Horror Movie, Oxford: Blackwell, 1989.

91 Brakhage ‘always enjoyed going to the movies, sometimes explaining the recreation as a means of staying in touch with the culture at large’. David E. James, ‘Introduction’, in James, op. cit. (72), pp. 1–19, 15.

92 See, for example, Elana Levine, Wallowing in Sex: The New Sexual Culture of 1970s American Television, Durham, NC: Duke University Press, 2007; Linda Williams, Screening Sex, Durham, NC: Duke University Press, 2008; and Eric Schaefer, Sex Scene: Media and the Sexual Revolution, Durham, NC: Duke University Press, 2014.

93 See Samantha Lay, British Social Realism: From Documentary to Brit-Grit, London: Wallflower, 2002; Jacob Leigh, The Cinema of Ken Loach: Art in the Service of the People, London: Wallflower, 2002; and Bigman, op. cit. (25).

94 See Waltraud Maierhofer, ‘Finding humor in birth control: fiction and film from Hugh Mills to Matthias Schweighhöfer’, in Maierhofer and Capo, op. cit. (49), pp. 136–155. Artificial insemination inspired *Test Tube Babies* (1948), an American exploitation film, and *A Question of Adultery* (1958), an X-rated British film based on the stage play *A Breach of Marriage* (1948), as well as more recent films. See Schaefer, op. cit. (50), pp. 206–208; and Peter Fitzpatrick, The Two Frank Thrings, Victoria: Monash University Publishing, 2012, pp. 401–403.

95 Solveig Jülich, ‘The making of a best-selling book on reproduction: Lennart Nilsson's *A Child Is Born*’, Bulletin of the History of Medicine (2015) 89, pp. 491–5252652167010.1353/bhm.2015.0091.

96 On evolution in *2001* see Carrol Fry, ‘From technology to transcendence: humanity's evolutionary journey in *2001: A Space Odyssey*’, Extrapolation (2003) 44, pp. 331–343; and Robert Poole, ‘*2001: A Space Odyssey* and the dawn of man’, in Tatjana Ljujic, Peter Kramer and Richard Daniels (eds.), Stanley Kubrick: New Perspectives, London: Black Dog Press, 2014, pp. 174–197.

97 Lucy Fischer, ‘Birth traumas: parturition and horror in *Rosemary's Baby*’, Cinema Journal (1992) 31, pp. 3–18; A. Robin Hoffman, ‘How to see the horror: the hostile fetus in *Rosemary's Baby* and *Alien*’, Literature Interpretation Theory (2011) 22, pp. 239–261; and Oliver, op. cit. (8), pp. 117–126.

98 On the grassroots campaign against *Z.P.G.* (1972), a science fiction film about overpopulation that seemed to promote the right to procreate at any cost to the environment, see Jesse Olszynko-Gryn and Patrick Ellis, ‘Malthus at the movies: science, cinema, and activism around *Z.P.G.* and *Soylent Green*’, *Cinema Journal*, forthcoming 2018. On *Histoires d'A* (1973), an initially banned ‘militant’ documentary about abortion that sparked protest at Cannes, see Romain Lecler, ‘Le succès d’*Histoires d'A*, “film sur l'avortement”: Une mobilisation croisée de ressources cinématographiques et militantes (enquête)’, Terrains & travaux (2007) 2, pp. 51–72; and Hélène Fleckinger, ‘*Histoires d'A*: Un moment de la lutte pour la liberté de l'avortement’, La revue documentaire (2010) 22–23, pp. 181–195.

99 On *Silent Scream* see Sara Dubow, Ourselves Unborn: A History of the Fetus in Modern America, Oxford: Oxford University Press, 2011, pp. 153, 159–160, 164. On IVF documentaries in the 1980s see Sarah Franklin, ‘Postmodern procreation: a cultural account of assisted reproduction’, in Faye D. Ginsburg and Rayna Rapp (eds.), *Conceiving the New World Order: The Global Politics of Reproduction*, Berkeley: University of California Press, 1995, pp. 323–345. Documentary footage of the birth of Louise Brown, the world's first child conceived by IVF, can be viewed at https://wellcomecollection.org/articles/first-test-tube-baby-louise-brown. See further Katie Dow, ‘Looking into the test-tube: the birth of IVF on British television’, in Jesse Olszynko-Gryn and Caroline Rusterholz (eds.), Reproductive Politics in France and Britain, a special issue of *Medical History*, forthcoming 2018.

100 Helen W. Robbins, ‘“More human than I am alone”: womb envy in David Cronenberg's *The Fly* and *Dead Ringers*’, in Steven Cohan and Ina Rae Hark (eds.), Screening the Male: Exploring Masculinities in Hollywood Cinema, London: Routledge, 1993, pp. 134–150.

101 Kate O'Riordan, ‘Human cloning in film: horror, ambivalence, hope’, Science as Culture (2008) 17, pp. 145–162. See further Joan Haran, Jenny Kitzinger, Maureen McNeil and Kate O'Riordan, Human Cloning in the Media: From Science Fiction to Science Practice, London: Routledge, 2007; and Sarah Franklin, Dolly Mixtures: The Remaking of Genealogy, Durham, NC: Duke University Press, 2007.

102 Amery Bodelson, ‘Redemptive restrooms: moments of utopic possibility in Volker Schlöndorff's film version of *The Handmaid's Tale*’, Journal of the Midwest Modern Language Association (2006) 39, pp. 63–72; Heather Latimer, “Bio-reproductive futurism: bare life and the pregnant refugee in Alfonso Cuarón's *Children of Men*’, Social Text (2011) 29, pp. 51–72; and Nicole L. Sparling, ‘Without a conceivable future: figuring the mother in Alfonso Cuarón's *Children of Men*’, Frontiers (2014) 35, pp. 160–180.

103 Leavitt, op. cit. (89), pp. 1–7; and Ziv Eisenberg, ‘The whole nine months: women, men, and the making of modern pregnancy in America’, PhD thesis, Yale University Press, 2013, pp. 262–331.

104 See Laura Mamo, Queering Reproduction: Achieving Pregnancy in the Age of Technoscience, Durham, NC: Duke University Press, 2007; and Jodi Brooks, ‘*The Kids Are All Right*, the pursuits of happiness, and the spaces between’, Camera Obscura (2014) 29, pp. 111–135. On earlier films about male pregnancy see JaneMaree Maher, ‘A pregnant man in the movies: the visual politics of reproduction’, Continuum (2008) 22, pp. 279–288; and Rodney Hill, ‘Queering the New-Wave deal: gender and sexuality in Jacques Demy's *A Slightly Pregnant Man*’, Post Script (2014) 34, pp. 50–60.

105 See Camilla A. Sears and Rebecca Godderis, ‘Roar like a tiger on TV? Constructions of women and childbirth in reality TV’, Feminist Media Studies (2011) 11, pp. 181–195; Letizia Guglielmo (ed.), MTV and Teen Pregnancy: Critical Essays on *16 and Pregnant* and *Teen Mom*, Lanham, MD: Rowan & Littlefield, 2013; Sofia Bull, ‘Midwives, medicine and natural births: female agency in Scandinavian birthing shows’, Critical Studies in Television (2016) 11, pp. 177–189; Tanya Horeck, ‘The affective labour of *One Born Every Minute* in its UK and US formats’, Critical Studies in Television (2016) 11, pp. 164–176; and Sara De Benedictis, ‘Watching *One Born Every Minute*: negotiating the terms of the “good birth”’, in Rachel Moseley, Helen Wheatley and Helen Wood (eds.), Television for Women: New Directions, New York: Routledge, 2017, pp. 110–127.

106 Jeffrey K. Ruoff, ‘Home movies of the avant-garde: Jonas Mekas and the New York art world’, in David E. James (ed.), To Free the Cinema: Jonas Mekas and the New York Underground, Princeton, NJ: Princeton University Press, 1992, pp. 294–312, 301. See further Segal, op. cit. (69).

107 See Robyn Longhurst, ‘YouTube: a new space for birth?’, Feminist Review (2009) 93, pp. 46–63.

108 Julie Roberts, ‘“Wakey wakey baby”: narrating four-dimensional (4D) bonding scans’, Sociology of Health & Illness (2012) 34, pp. 299–3142150701010.1111/j.1467-9566.2011.01345.x, 301. See further Roberts, The Visualised Foetus: A Cultural and Political Analysis of Ultrasound Imagery, Farnham: Ashgate, 2012.

109 See Sarah Franklin, Biological Relatives: IVF, Stem Cells, and the Future of Kinship, Durham, NC: Duke University Press, 2013, pp. 246–254; and Lucy van de Wiel, ‘Cellular origins: a visual analysis of time-lapse embryo imaging’, in Merete Lie and Nina Lykke (eds.), Assisted Reproduction across Borders: Feminist Perspectives on Normalizations, Disruptions and Transmissions, New York: Routledge, 2017, pp. 288–301.

110 See Brian M. Stramer and Graham A. Dunn, ‘Cells on film: the past and future of cinemicroscopy’, Journal of Cell Science (2015) 128, pp. 9–132555624910.1242/jcs.165019.

111 See Jacqueline H. Wolf, ‘Film as the medium; reproduction, sex, and power as the message’, Journal of Women's History (2010), 22, pp. 173–18410.1353/jowh.2010.059220857597.

112 For example, Debra Pascali-Bonaro's *Orgasmic Birth: The Best-Kept Secret* (2009) and Rebecca Haimowitz and Vaishali Sinha's *Made in India: A Film about Surrogacy* (2010). Irene Lusztig's *The Motherhood Archives* (2013) makes the most effective use of archival footage, including from recently unearthed Soviet and French childbirth films; see http://motherhoodarchives.net. Earlier feminist documentaries notably interrogated coercive sterilization practices in Puerto Rico and India: Kimberly Safford, ‘*La Operación*: forced sterilization’, Jump Cut (1984) 29, pp. 37–38; and Madhumeeta Sinha, ‘Witness to violence: documentary cinema and the women's movement in India’, Indian Journal of Gender Studies (2010) 17, pp. 365–373. Certain of these films and many others are available from Women Make Movies at www.wmm.com/index.asp.

